# Developmental Plasticity of the Major Alkyl Cannabinoid Chemotypes in a Diverse *Cannabis* Genetic Resource Collection

**DOI:** 10.3389/fpls.2018.01510

**Published:** 2018-10-23

**Authors:** Matthew T. Welling, Lei Liu, Carolyn A. Raymond, Omid Ansari, Graham J. King

**Affiliations:** ^1^Southern Cross Plant Science, Southern Cross University, Lismore, NSW, Australia; ^2^Ecofibre Industries Operations Pty Ltd., Brisbane, QLD, Australia; ^3^Ananda Hemp Ltd., Cynthiana, KY, United States

**Keywords:** *Cannabis sativa* L., hemp, medicinal *Cannabis*, LC-MS, propyl alkyl cannabinoids, tetrahydrocannabivarinic acid, cannabidivarinic acid

## Abstract

*Cannabis* is a chemically diverse domesticated plant genus which produces a unique class of biologically active secondary metabolites referred to as cannabinoids. The affinity and selectivity of cannabinoids to targets of the human endocannabinoid system depend on alkyl side chain length, and these structural-activity relationships can be utilized for the development of novel therapeutics. Accurate early screening of germplasm has the potential to accelerate selection of chemical phenotypes (chemotypes) for pharmacological exploitation. However, limited attempts have been made to characterize the plasticity of alkyl cannabinoid composition in different plant tissues and throughout development. A chemotypic diversity panel comprised of 99 individuals from 20 *Cannabis* populations sourced from the Ecofibre Global Germplasm Collection (ecofibre.com.au and anandahemp.com) was used to examine alkyl cannabinoid variation across vegetative, flowering and maturation stages. A wide range of di-/tri-cyclic as well as C_3_-/C_5_-alkyl cannabinoid composition was observed between plants. Chemotype at the vegetative and flowering stages was found to be predictive of chemotype at maturation, indicating a low level of plasticity in cannabinoid composition. Chemometric cluster analysis based on composition data from all three developmental stages categorized alkyl cannabinoid chemotypes into three classes. Our results suggest that more extensive chemical and genetic characterization of the *Cannabis* genepool could facilitate the metabolic engineering of alkyl cannabinoid chemotypes.

## Introduction

*Cannabis sativa* L. is the sole, formally recognized species within the genus *Cannabis* and is a member of the angiosperm family Cannabaceae (Small and Cronquist, 1976). *Cannabis* is diploid (Van Bakel et al., 2011), predominately dioecious, and obligate outbred (Faeti et al., 1996) and can be considered highly heterozygote (Soler et al., 2017). The extant genepool is comprised principally of domesticated or previously domesticated feral populations (Welling et al., 2016b), with intraspecific groupings based on selection of phenotypes primarily associated with seed/fiber (industrial hemp), recreational drug (marijuana) (Mandolino and Carboni, 2004) and, more recently, therapeutic end-uses (Potter, 2014).

The predominant bioactive secondary metabolites produced by *Cannabis* are the terpenophenolic phytocannabinoids (cannabinoids), of which >100 have been identified (ElSohly and Slade, 2005; Radwan et al., 2015). Structurally related terpenophenolic compounds also occur in other plant species such as the prenylflavonoids in *Humulus lupulus* (Stevens et al., 1999), a closely related species within the Cannabaceae which is thought to have diverged ∼21 MYA (Divashuk et al., 2014). However, the cannabinoids appear largely unique to *Cannabis* (Gertsch et al., 2010), and are formed at high concentrations within capitate stalked trichomes on the floral tissues of female inflorescences. They also accumulate within capitate-sessile trichomes and potentially bulbous trichomes on floral as well as non-floral tissues including leaves and stems (Happyana et al., 2013). Despite their relative abundance and interspersed distribution in plant tissue, the metabolic role of cannabinoids in *Cannabis* is largely unknown, although they may mitigate biotic stress via mitochondrial membrane dysfunction-induced necrosis in leaf cells (Morimoto et al., 2007).

Cannabinoids are produced in *Cannabis* in their carboxylic acid (COOH) forms and are decarboxylated to neutral cannabinoids in a non-enzymatic reaction which can be accelerated at temperatures >100°C (Dussy et al., 2005). Decarboxylation can also occur after extended periods of storage >100 days at room temperature (Hanuš et al., 2016). A notable example of this is the conversion of the non-psychoactive delta(9)-tetrahydrocannabinolic acid (THCA) to the psychoactive delta(9)-tetrahydrocannabinol (THC) (Izzo et al., 2009) upon loss of the COOH group.

**FIGURE 1:**
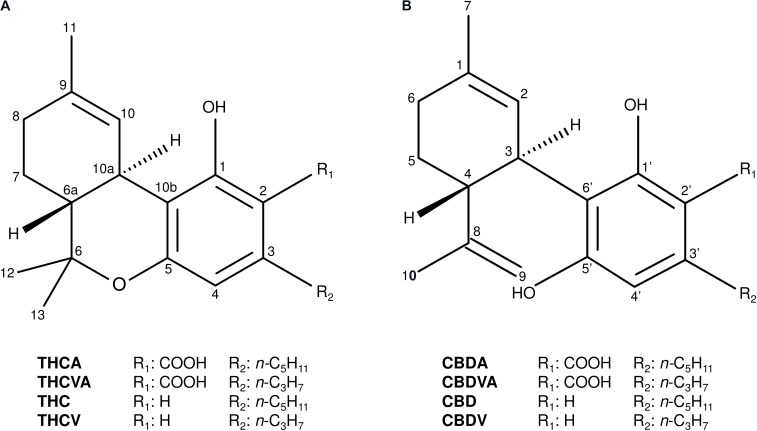
Chemical structures of the major tricyclic and dicyclic alkyl cannabinoids in *Cannabis*. **(A)** Tricyclic cannabinoids. **(B)** Dicyclic cannabinoids. Cannabidiol (CBD); cannabidiolic acid (CBDA); cannabidivarin (CBDV); cannabidivarinic acid (CBDVA); delta(9)-tetrahydrocannabinol (THC); delta(9)-tetrahydrocannabinolic acid (THCA); delta(9)-tetrahydrocannabivarin (THCV); and delta(9)-tetrahydrocannabivarinic acid (THCVA).

The tricyclic THCA and dicyclic cannabidiolic acid (CBDA) C_5_-alkyl cannabinoids are the most predominant and commonly occurring cannabinoids in *Cannabis* (Figure 1; Hazekamp et al., 2016). A series of C_3_-alkyl cannabinoid homologs, including the tricyclic delta(9)-tetrahydrocannabivarinic acid (THCVA) and dicyclic cannabidivarinic acid (CBDVA), can also contribute significantly to the cannabinoid profiles of ecotypes from Asian (Figure 1; Hillig and Mahlberg, 2004; Welling et al., 2016a) and African provenance (Baker et al., 1983), although these compounds are typically found at low levels in contemporary domesticated forms (Swift et al., 2013; Hazekamp et al., 2016; Welling et al., 2016a). Trace amounts of other alkyl homologs have also been identified such as methyl-(C_1_) (Vree et al., 1972) and butyl-(C_4_) (Smith, 1997) alkyl cannabinoids, although accounts of high levels of these cannabinoids *in planta* are scarce.

Current understanding of the bioactivity of cannabinoids is based on their modulation of the human endocannabinoid system, a poorly defined complex ensemble of several receptors, two endogenous cannabinoid ligands *N*-arachidonoylethanolamine (anandamide) and 2-arachidonoylglycerol (2-AG) as well as associated enzymatic pathways (Di Marzo and Piscitelli, 2015). The cannabinoid alkyl side chain is a critical pharmacophore (Khanolkar et al., 2000), with changes in carbon length influencing the affinity and selectivity of plant derived cannabinoids to targets of the human endocannabinoid system (Thakur et al., 2005). Indeed, recent docking studies using a 2.6-Å resolution crystal structure of the human G-protein-coupled cannabinoid type-1 receptor (CB_1_R) show binding of the tricyclic core of THC with a number of transmembrane domains preceding a highly conserved membrane-proximal N-terminal region, with the alkyl side chain extending toward a Trp356^6.48^ residual (Shao et al., 2016) associated with CB_1_R activation (Shim et al., 2011). Subsequent partial agonist binding by THC to CB_1_R stimulates mesolimbic dopamine activity (French, 1997), a mechanism believed to be partially responsible for this ligands psychoactivity.

Until recently, plant cannabinoids have primarily seen use in the context of recreational drug use of THC. However, they offer promise as novel therapeutics in a number of diverse non-communicable diseases. The company GW Pharmaceuticals, plc has developed cannabidiol (CBD) and THC containing Sativex^®^ (Chandra et al., 2017), a prescription medicine approved for the management of multiple sclerosis in more than 22 countries^1^, as well as CBD containing Epidiolex^®^ which has recently been approved by the US Food and Drug Administration (FDA) for the treatment of childhood seizures associated with Lennox-Gastaut syndrome and Dravet syndrome (Chandra et al., 2017). Ananda Hemp Ltd. (a subsidiary company of Ecofibre Industries Operations Pty Ltd.) has recently launched a range of cannabinoid-based products^2^. The C_3_-alkyl cannabinoids cannabidivarin (CBDV) and delta(9)-tetrahydrocannabivarin (THCV) are also emerging as therapeutic entities. CBDV has been targeted by GW Pharmaceuticals, plc (Vemuri and Makriyannis, 2015), with phase I and II clinical trials having been initiated for the treatment of autism spectrum disorders and epilepsy, respectively. Moreover, a double-blind, placebo-controlled pilot study of 62 non-insulin treated type II diabetes subjects supports a therapeutic role for THCV in the modulation of fasting blood glucose and pancreatic β-cell function (Jadoon et al., 2016).

Current methods for the production of cannabinoid-based botanical drug products rely predominantly on clonal propagation of plants (Lata et al., 2012) due to the limited ability to predict chemical heritability in seed propagated progeny (Potter, 2014). Development of early diagnostic techniques to determine C_3_-alkyl cannabinoid quality (CBDV + THCV) within the total cannabinoid fraction could assist breeders in the selection of elite alkyl cannabinoid breeding lines. While the ontogenetic variation in di-/tri-cyclic cannabinoid composition during plant development within the C_5_-alkyl cannabinoid fraction has been studied (Pacifico et al., 2008; De Backer et al., 2012; Aizpurua-Olaizola et al., 2016; Richins et al., 2018), there have been limited attempts to characterize developmental changes of C_3_-alkyl cannabinoid composition. Moreover, alkyl cannabinoid chemotypes have not been systematically evaluated among divergent subtaxa.

This lack of clarity in understanding the extent to which alkyl cannabinoid composition varies *in planta* limits the ability to use chemotypic assessment during early developmental stages as well as to predict chemotype prior to seed formation. In the present study, liquid chromatography-mass spectrometry (LC-MS) profiling of a chemotypic diversity panel with a representative range of genotypes within the *Cannabis* genepool was used to characterize variation in alkyl cannabinoid composition across vegetative, flowering and maturation stages. Seed-based accessions were sourced from the Ecofibre Global Germplasm Collection with priority given to accessions with provenance from Southern, Eastern and Western Asia as well as Africa to ensure adequate representation of C_3_-alkyl cannabinoid chemotypes (Hillig and Mahlberg, 2004; Welling et al., 2016a; Table 1). Cluster analysis of alkyl cannabinoid fractions was performed to provide insight into the categorization and genetic regulation of alkyl cannabinoid chemotypes in *Cannabis*.

**Table 1:** Description of 20 *Cannabis* accessions used for alkyl cannabinoid chemotypic characterization across three developmental stages.

Accession	ID	Individuals (*n*)	Provenance	Taxon	Source
EIO.MW15.A	A	3	Southern Asia	*Cannabis sativa* L.	EFGGC
EIO.MW15.B	B	4	Eastern Asia	*Cannabis sativa* L.	EFGGC
EIO.MW15.C	C	5	Eastern Asia	*Cannabis sativa* L.	EFGGC
EIO.MW15.D	D	3	Eastern Asia	*Cannabis sativa* L.	EFGGC
EIO.MW15.E	E	5	Eastern Asia	*Cannabis sativa* L.	EFGGC
EIO.MW15.F	F	5	Eastern Asia	*Cannabis sativa* L.	EFGGC
EIO.MW15.G	G	6	Eastern Asia	*Cannabis sativa* L.	EFGGC
EIO.MW15.I	I	6	Southern Asia	*Cannabis sativa* L.	EFGGC
EIO.MW15.J	J	6	Eastern Asia	*Cannabis sativa* L.	EFGGC
EIO.MW15.K	K	4	Eastern Asia	*Cannabis sativa* L.	EFGGC
EIO.MW15.L	L	4	Eastern Asia	*Cannabis sativa* L.	EFGGC
EIO.MW15.M	M	7	Eastern Asia	*Cannabis sativa* L.	EFGGC
EIO.MW15.O	O	6	Eastern Asia	*Cannabis sativa* L.	EFGGC
EIO.MW15.P	P	6	Eastern Asia	*Cannabis sativa* L.	EFGGC
EIO.MW15.Q	Q	5	Caribbean	*Cannabis sativa* L.	EFGGC
EIO.MW15.R	R	6	Southern Asia	*Cannabis sativa* L.	EFGGC
EIO.MW15.S	S	3	Southern Africa	*Cannabis sativa* L.	EFGGC
EIO.MW15.T	T	5	Western Asia	*Cannabis sativa* L.	EFGGC
EIO.MW15.U	U	7	Eastern Africa	*Cannabis sativa* L.	EFGGC
EIO.MW15.X	X	3	Eastern Asia	*Cannabis sativa* L.	EFGGC


*EFGGC, Ecofibre Global Germplasm Collection.*

## Materials and Methods

### Genetic Resources

Acquisition, storage and experimental endeavors were performed under the provisions of the Drug Misuse and Trafficking Act 1985 and in accordance with authorizations granted by the New South Wales Ministry of Health, Pharmaceutical Regulatory Unit, Legal and Regulatory Services Branch, Australia. Seed accessions were obtained from the Ecofibre Global Germplasm Collection owned by the company Ecofibre Industries Operations Pty Ltd. and managed by Southern Cross University, Australia. A single seed pack accession in a *Cannabis* genetic resource base collection can be generated from multiple parents and so is provisionally considered as a population (Faeti et al., 1996). Twenty populations (accessions) with geographical origins associated with C_3_-alkyl cannabinoid accumulation (Hillig and Mahlberg, 2004) were preferentially selected to ensure an adequate level of alkyl cannabinoid chemotypic diversity (Table 1).

### Growth Parameters

Growth parameters followed those of Welling et al. (2016a). Seeds were planted at a depth of 1.5 cm in cells of 5 cm (diameter) × 6 cm (height) in a mix of one part vermiculite, one part perlite, peat moss, and dolomite (110g/100L). CANNA^®^ Aqua Vega nutrient solution was used as a supplement. Seedling trays (40 cells) were watered with 500 mL of water three times per day for 14 days. Seedlings were transplanted to 8 L pots, with each pot containing 8 g of Micromax^®^ micronutrient formula and 100 g Osmocote^®^ Exact slow release nutrient mix. Plants were grown in chambers fitted with ‘smart valves’ to maintain optimal water regimes. Temperature was maintained between 26 and 28°C, and plants were subject to 11 h of high pressure sodium (HPS)/metal halide (MH) light (luminous flux = 72,000 lumens) per day.

A total of 99 individual female plants were chemotyped at three developmental stages, with three to seven plants analyzed per accession (Table 1). Developmental stages were determined from visual inspection of plant morphological changes defined in the Decimal Code for Growth Stages of Hemp (Mediavilla et al., 1998). Two × 250 mg fresh plant material was collected from the sub-apical raceme of each individual at opposing phyllotaxis during vegetative (fourth leaf pair, code 1008) and alternate phyllotaxis during flowering (code 2202) stages. Fresh leaf material was snap-frozen using liquid nitrogen in 2 mL Eppendorf^®^ Safe-Lock microcentrifuge tubes and stored at -80°C. At seed maturation (code 2202) individual plant racemes were dried at 35°C in a forced ventilation oven for 72 h and stored at room temperature in air sealed containers with 3–5 mm orange silico gel beads.

### Sample Preparation and Extraction

Disruption of fresh leaf tissue was performed using a Qiagen TissueLyser^®^. Frozen leaf tissue was ground in a 2 mL Eppendorf^®^ Safe-Lock microcentrifuge tube containing a 3 mm Qiagen Tungsten Carbide Bead (Cat No./ID: 69997). Microcentrifuge tubes were agitated at 30 rotations per sec for 2 × 30 s intervals. Tissue was extracted in 1 mL of high-performance liquid chromatography (HPLC) grade EtOH (100%). Extractions were vortexed and mixed by agitation for 30 min. To remove particulate material, samples were centrifuged using a Compact centrifuge 2–5 (Sigma 113) at 8000 rpm for 10 min. The supernatant (600 μL) was transferred into a 2 mL screw cap glass vial and subject to a 1:5 dilution to ensure signals were within calibration range.

Sample preparations for dried leaf tissue followed those of De Backer et al. (2009) and Welling et al. (2016a) with slight modification. Dried leaf tissue was ground with a Mixer Mill MM 301 (Retsch GmbH) at 30 rotations per sec for 30 s intervals. Duplicate extracts were performed for each plant per accession. Approximately 250 mg of dried leaf tissue was extracted in 25 mL of HPLC grade EtOH (100%) for 30 min. To remove particulate material, 1 mL of the extract was centrifuged using a Compact centrifuge 2–5 (Sigma) at 3000 rpm for 10 min. The supernatant (600 μL) was transferred into 2 mL screw cap glass vial and all samples were subject to a 1:5 dilution to ensure signals were within calibration range.

### LC-MS Cannabinoid Profiling

Liquid chromatography-mass spectrometry (LC-MS) cannabinoid profiling runs were conducted using an Agilent 1290 Infinity analytical HPLC instrument (Agilent Technologies, Palo Alto, CA, United States), comprising of a vacuum degasser, autoinjector, binary pump and diode array detector (DAD, 1260), coupled with an Agilent 6120 Single Quadrupole mass detector (MSD). The LC-MS instrument was controlled using Agilent ChemStation software (Rev. B.04.03 [54]). Absorbance was monitored at 210 nm, 214 nm, 272 nm, 280 nm, 330 nm and 360 nm. An Agilent Eclipse plus rapid resolution high definition (RRHD) C_18_ column (1.8 μm; 50 mm × 2.1 mm internal diameter) was used and column temperature was set at 30°C. Injection volume was 3 μL.

The mobile phase followed those of Giese et al. (2015) with minor modification. Mobile phases consisted of 0.005% TFA in Milli-Q^®^ water for channel A and 0.005% TFA in acetonitrile for channel B. Flow rate was 0.3 mL/min starting with a isocratic phase at 66% B for 8 min, then a linear gradient to 95% B over 4 min. 95% B was held for 1 min, then re-equilibrated to 66% B for 1 min. Equilibration was further extended for 1 min to perform an internal needle wash of the autosampler to minimize carryover. Run time was 16 min.

MSD parameters followed those of Liu et al. (2014) and Welling et al. (2016a) with modification to allow quantification of four additional cannabinoids; THCVA, CBDVA, CBDV and cannabichromene (CBC). The MSD was operated in atmospheric pressure electrospray ionization mode (AP-ESI); scan mass range, 100-1200; drying gas temperature, 350°C; fragmentor, 150; capillary voltage, 3000 V (positive); vaporizer temperature, 350°C; drying gas flow, 12 L/min (N_2_); nebulizer pressure, 35 psi.

Quantification of cannabinoids was performed using selected-ion monitoring (SIM) with four available MSD signal channels (Supplementary Table S1). THCA, THC, THCV, cannabinol (CBN), cannabigerolic acid (CBGA), cannabigerol (CBG), CBDA, CBD, CBDV, and CBC cannabinoid standards were sourced from Novachem Pty Ltd. (Melbourne, VIC, Australia). THCVA and CBDVA were isolated from plant tissue to develop analytical standards. All cannabinoid reference standards were scanned in positive mode [M + H]^+^ to determine the most abundant and representative signal.

Quadratic regression of calibration curves of individual reference standards was used to determine cannabinoid concentrations. Calibration curves were obtained from six solutions comprising of five acid cannabinoid standards THCA, CBDA, CBGA, THCVA, and CBDVA at the following concentrations; 0.032, 0.16, 0.8, 4, 20, and 100 μg/mL. Calibration curves were also obtained from six solutions comprising of seven neutral cannabinoid standards THC, THCV, CBN, CBG, CBD, CBDV, and CBC at the following concentrations; 0.032, 0.16, 0.8, 4, 20, and 100 μg/mL. Linear regression analysis showed calibration curves to be linear within the concentration range for each cannabinoid (*R*^2^ > 0.99). To minimize MSD interday variability, calibration curves were performed daily. The precision of the MSD was examined by injecting standard solutions six times within a 24 h period and relative standard deviation (RSD) for each cannabinoid peak area was <2%.

### Statistical Analysis

To test for repeatability between extraction replicates, the C_3_-alkyl (F_C3_), C_5_-alkyl (F_C5_)_,_ dicyclic (F_dicyclic_), and tricyclic (F_tricyclic_) cannabinoid fractions were calculated using *R*^2^. Strong positive correlations between extraction replicates were found for the F_C3_/F_C5_ values (*R^2^* > 0.99) as well as for the F_dicyclic_/F_tricyclic_ values (*R^2^* > 0.99) at vegetative, flowering and maturation stages. As such, mean values gathered from duplicate extraction replicates were utilized for statistical analysis. Statistical analysis was performed using GenStat 64-bit Release 18.1 (VSN International Ltd.) software. For regression analysis, the constant (intercept) was omitted and the fitted line was constrained through the origin. For non-hierarchical *k*-means cluster analysis, similarities were calculated using Euclidean distance.

### Isolation, Purification, and Structural Elucidation of C_3_-Alkyl Cannabinoids

Dried female *Cannabis* floral tissue (4 × 1 g) sourced from the Ecofibre Global Germplasm Collection was extracted in 100% MeOH (4 × 20 mL) and evaporated using a Christ^®^ BETA- RVC rotational vacuum concentrator. Extracts were pooled, resuspended in MeOH (4 mL) and partitioned using *n*-hexane (4 mL) to remove chlorophyll. The MeOH fraction was separated using a glass pipette, centrifuged to remove particulate matter and evaporated using a Christ^®^ BETA- RVC rotational vacuum concentrator. The crude MeOH fraction (486 mg) was then resuspended in 6:4 MeOH: Milli-Q^®^ water (2 mL).

Isolation and purification of the crude *Cannabis* MeOH extract was performed using an Agilent 1260 Infinity preparative HPLC system, comprising of a vacuum degasser, autosampler, binary preparative pump, diode array detector (DAD, 1260) and analytical-scale fraction collector. The preparative HPLC instrument was controlled using Agilent ChemStation software (Rev. B.04.03 [16]). Absorbance was monitored at 210 nm, 254 nm, 272 nm, 280 nm and 360 nm. A Luna C_18_ column (5 μm; 150 mm × 21.20 mm internal diameter) was used. Injection volume was 500 μL. Mobile phases consisted of 0.05% TFA in Milli-Q^®^ water for channel A and 0.05% TFA in acetonitrile for channel B. Flow rate was 20 mL/min, starting with a isocratic phase at 80% B for 3 min, then a linear gradient to 99% B over 5 min. 99% B was held for 5 min, then re-equilibrated to 80% B for 2 min and held at 80% B for 5 min. Run time was 20 min. The fraction collector was operated in time-based trigger mode at 0.18 min time slices. THCVA (1.57 mg) and CBDVA (1.83 mg) fractionations were evaporated using a Christ^®^ BETA- RVC rotational vacuum concentrator and redissolved in HPLC grade EtOH (100%).

Structural elucidation of C_3_-alkyl cannabinoids THCVA and CBDVA was performed using a Bruker Avance III HDX 800 MHz spectrometer. LC-MS spectra were obtained using an Agilent 1290 Infinity analytical HPLC instrument (Agilent Technologies, Palo Alto, CA, United States), comprising of a vacuum degasser, autoinjector, binary pump and diode array detector (DAD, 1260), coupled with an Agilent 6120 Single Quadrupole MSD. UV spectra were monitored at 210, 272, 280 and 360. For two dimensional NMR, ^1^H-^1^H Correlation Spectroscopy (^1^H-^1^H-COSY), Heteronuclear Single Quantum Coherence (HSQC), Heteronuclear Multiple Bond Correlation (HMBC), and Rotating-Frame Overhauser Spectroscopy (ROESY) experiments were performed. Data analysis, acquisition and processing of NMR and LC-MS spectra was conducted using TopSpin^TM^ (TS3.5pl6) and Agilent ChemStation© (Rev. B.04.03 [54]) software, respectively.

## Results

### Structural Elucidation of Acidic C3-Alkyl Cannabinoids

**FIGURE 2:**
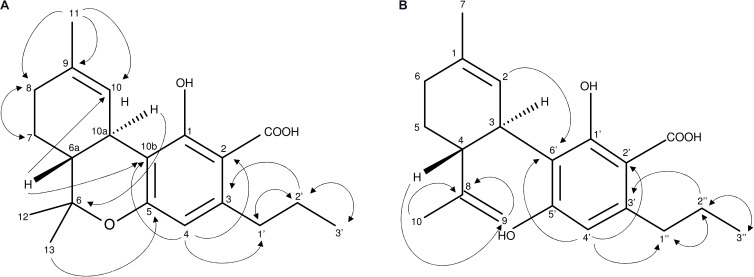
**(A,B)** Important ^1^H-^1^H-COSY and HMBC NMR correlations of compounds **1** **(A)** and **2** **(B)** describing the C_3_-alkyl side chain of **1** **(A)** and **2** **(B)** as well as the opened pyran ring of **2**.

At the time of analysis, analytical standards for THCVA and CBDVA were not commercially available. Unknown compounds **1** and **2** were isolated and purified from *Cannabis* floral tissue, with structural elucidation performed using LC-MS (Supplementary Figures S1, S2) as well as ^1^H (Supplementary Figures S3, S4) ^13^C NMR (Supplementary Figures S5, S6) and 2D NMR (Supplementary Figures S7–S14). AP-ESI MS spectra of **1** and **2** exhibited the expected molecular ion *m/z* 328.9 [M-H]^-^ (calculated for C_20_H_26_O_4_, 330.42). Positioning of the C_3_-alkyl side chain at C-3 of **1** and C-3′ of **2** as well as the opened pyran ring configuration of **2** between C-8 and C-5′ were confirmed from ^1^H-^1^H-COSY (Supplementary Figures S7, S8) and HMBC (Supplementary Figures S11, S12) NMR spectra (Figure 2). The presence of signals δ_C_ 173.9 (2-COOH) (**1**) and δ_C_ 174.2 (2′-COOH) (**2**) (Supplementary Figures S5, S6) as well as the absence of a -OH group at associated positions was characteristic of a COOH at C-2 of **1** and C-2′ of **2**, which confirmed that both compounds were acidic cannabinoids. The ROESY spectrum suggested a *trans* relationship between H-6a and H-10a of **1** as well as H-4 and H-3 of **2** (Supplementary Figures S13, S14). Compounds **1** and **2** were subsequently defined as THCVA and CBDVA, respectively.

### Distribution of the Major Cyclic and Alkyl Cannabinoid Chemotypes

Chemotypes of 99 individual *Cannabis* plants from 20 seed accessions were characterized across three developmental stages using LC-MS analysis. Fresh leaf tissue samples were taken at the vegetative and flowering stages and cannabinoid composition was compared with dried floral tissue cannabinoid composition at maturation. The dicyclic cannabinoids cannabichromenic acid (CBCA) and cannabichromevarinic acid (CBCVA) as well as the precursor C_3_-alkyl cannabinoid cannabigerovarinic acid (CBGVA) were not commercially available at the time of analysis, nor were these compounds present at sufficient quantities to develop analytical standards. THCA, CBDA, THCVA, and CBDVA as well as corresponding neutral decarboxylated derivatives were used as a proxy for C_3_-alkyl (F_C3_) and C_5_-alkyl (F_C5_) as well as dicyclic (F_dicyclic_) and tricyclic (F_tricyclic_) cannabinoid fractions within the total cannabinoid fraction. Calculation of the total cannabinoid fraction was achieved by the addition of THCA, CBDA, THCVA, and CBDVA as well as their neutral cannabinoids (Supplementary Table S2). To determine the total cannabinoid fraction and to compare the F_C3,_ F_C5,_ F_dicyclic_, and F_tricyclic_ values between juvenile and mature plants, neutral cannabinoids CBDV, CBD, THCV, and THC were expressed as acidic cannabinoids using formulae which accounted for differences in molecular weight:

$$F_{C3}[\%]=\frac{\left((THCVA+CBDVA)+((THCV+CBDV)\times 1.1536)\right)}{total}\times 100$$

$$F_{\mathrm{C5}}[\%]=\frac{\left((THCA+CBDA)+((THC+CBD)\times 1.1399)\right)}{total}\times 100$$

$$F_{\mathrm{dicyclic}}[\%]=\frac{\left((CBDVA+CBDA)+((CBDV\times 1.1536)+(CBD\times 1.1399))\right)}{total}\times 100$$

$$F_{\mathrm{tricyclic}}[\%]=\frac{\left((THCVA+THCA)+((THCV\times 1.1536)+(THC\times 1.1399))\right)}{total}\times 100$$

**FIGURE 3:**
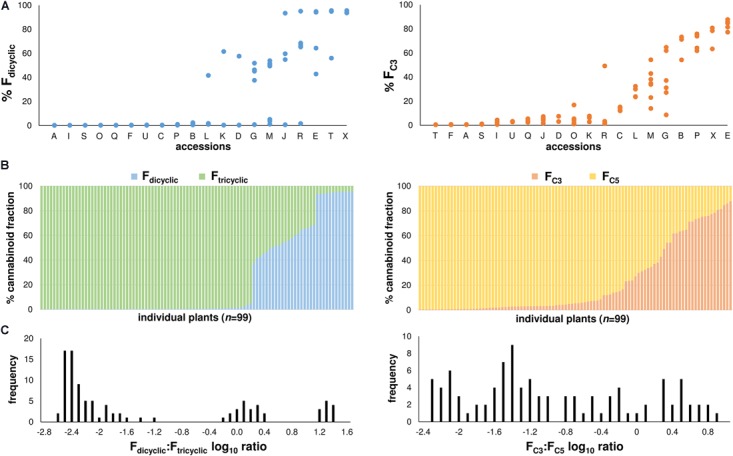
**(A)** F_dicyclic_ as well as F_C3_ chemotypic variation of mature plants within accessions. Accessions ordered on the *x*-axis from low to high chemotypic values. F_dicyclic_ as well as F_C3_ values on the *y*-axis describe the relative abundance of dicyclic as well as C3-alkyl cannabinoid fractions. *Letters* specify accession ID (Table 1). **(B)** Distribution patterns of the major F_dicyclic_/F_tricyclic_ as well as F_C3_/F_C5_ values of 99 *Cannabis* plants at maturation. Individual plants ordered on the x-axis from low to high F_dicyclic_ as well as F_C3_ chemotypic values. F_dicyclic_/F_tricyclic_ as well as F_C3_/F_C5_ values on the *y*-axis describe the relative abundance of dicyclic as well as C3-alkyl cannabinoid fractions. **(C)** F_dicyclic_: F_tricyclic_ as well as F_C3_: F_C5_ log10 ratios of 99 mature *Cannabis* plants. Log10 frequency distributions of F_dicyclic_: F_tricyclic_ chemotypic values show three discrete distributions, while Log10 frequency distributions of F_C3_: F_C5_ chemotypic values have no obvious distribution pattern; C_5_-alkyl cannabinoid fractions (F_C5_); C_3_-alkyl cannabinoid fractions (F_C3_); dicyclic cannabinoid fractions (F_dicyclic_); and tricyclic cannabinoid fractions (F_tricyclic_).

At maturation, variation in chemotype appeared to segregate within the accessions and so chemotype was reported at the plant level (Figure 3A), although within-accession chemotypic variation was more evident from the F_dicyclic_ values than from the F_C3_ values (Figure 3A). Distributions of the di-/tri-cyclic as well as the C_3_-/C_5_-alkyl cannabinoid fractions at maturation were skewed toward high F_tricyclic_ and F_C5_ values, respectively (Figure 3B). A wide range of the di-/tri-cyclic as well as the C_3_-/C_5_-alkyl cannabinoid fractions was found within the chemotypic diversity panel derived from the Ecofibre Global Germplasm Collection, with F_C3_ values ranging from 0.43% (±0.00%) to 87.78% (±0.10%) (Figure 3B). Plants from the Ecofibre accessions E as well as P (Ecofibre proprietary line) had the highest proportions of dicyclic (CBDVA) and tricyclic (THCVA) C_3_-alkyl cannabinoids, respectively. The plant from accession E with the highest dicyclic C_3_-alkyl cannabinoid fraction exhibited 81.2% CBDVA (% total cannabinoids), while the plant from accession P (Ecofibre proprietary line) with the highest tricyclic C_3_-alkyl cannabinoid fraction exhibited 75.1% THCVA (% total cannabinoids). Three discrete distributions comprised of low F_dicyclic_: F_tricyclic_, intermediate F_dicyclic_: F_tricyclic_, and high F_dicyclic_: F_tricyclic_ ratios were observed (Figure 3C), while the C_3_-/C_5_-alkyl cannabinoid proportions/ratios presented as a continuum with no obvious distribution patterns (Figures 3B,C).

### Stability of Alkyl Cannabinoid Composition

**FIGURE 4:**
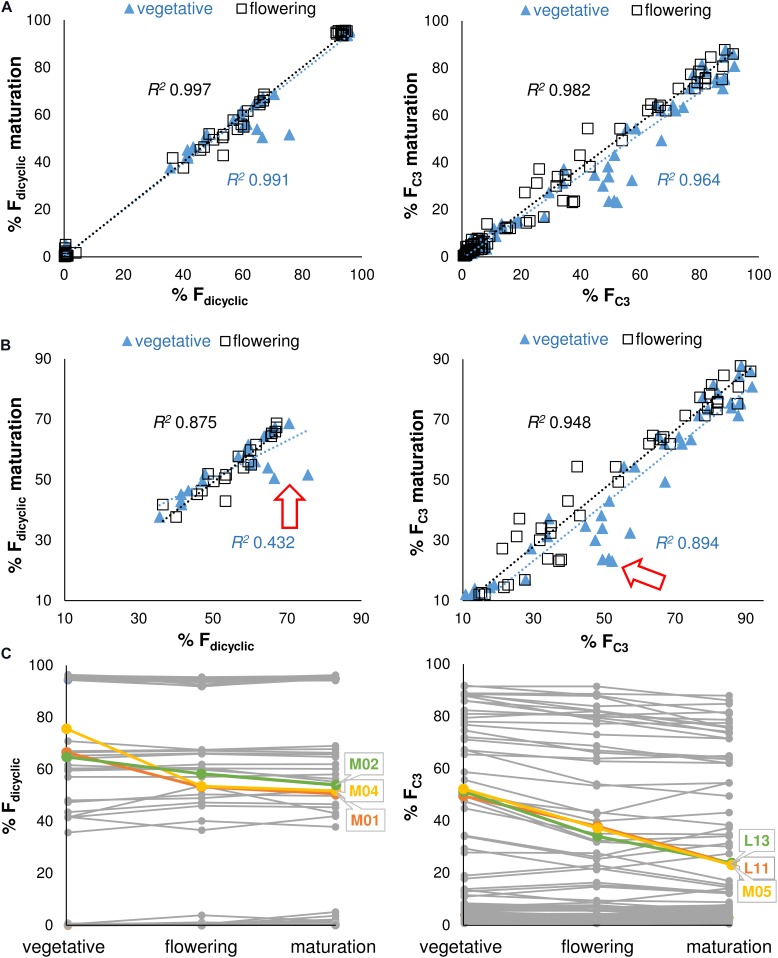
**(A)** Regression analysis of the F_dicyclic_ as well as the F_C3_ chemotypic values between developmental stages. F_dicyclic_ as well as the F_C3_ values describe dicyclic as well as C3-alkyl cannabinoid fractions. The F_dicyclic_ as well as the F_C3_ chemotypic values on the *x*-axis describe cannabinoid fractions at the vegetative and flowering stages. **(B)** Regression analysis of the truncated F_dicyclic_ as well as F_C3_ values between developmental stages. The F_dicyclic_ as well as the F_C3_ chemotypic values on the *x*-axis describe dicyclic as well as C3-alkyl cannabinoid fractions at the vegetative and flowering stages. **(C)** Individual plants with large standardized residuals across vegetative and maturation growth stages. The F_dicyclic_ as well as the F_C3_ chemotypic values on the *y*-axis describe dicyclic as well as C3-alkyl cannabinoid fractions across developmental stages. *Red arrow* indicates position of units with large standardized residuals; *Letters* specify accession ID (Table 1); *Numbers* indicate plant individual within accession; C_5_-alkyl cannabinoid fractions (F_C5_); C_3_-alkyl cannabinoid fractions (F_C3_); dicyclic cannabinoid fractions (F_dicyclic_); and tricyclic cannabinoid fractions (F_tricyclic_).

A simple linear regression model was calculated to predict the di-/tri-cyclic as well as the C_3_-/C_5_-alkyl cannabinoid fractions at maturation based on cannabinoid fractions at vegetative and flowering stages. Regressions were significant at the vegetative stage for the F_dicyclic_ values [*F*(1, 98) = 15772.31, *p* < 0.001], with an *R^2^* 0.991, as well as for the F_C3_ values [*F*(1, 98) = 4301.82, *p* < 0.001], with an *R^2^* > 0.964 (Figure 4A). Cannabinoid fractions showed minimal plasticity throughout development, with significant regressions also found at the flowering stage for the F_dicyclic_ values [*F*(1, 98) = 50480.89, *p* < 0.001], with an *R^2^* 0.997, as well as for the F_C3_ values [*F*(1, 98) = 8488.54, *p* < 0.001], with an *R^2^* > 0.982 (Figure 4A).

As the di-/tri-cyclic as well as the C_3_-/C_5_-alkyl cannabinoid fractions approached parity in the vegetative stage, they appeared less predictive of chemotype at maturation when compared with cannabinoid fractions at the flowering stage (Figure 4A). To examine this further we truncated the F_dicyclic_ (*n* = 20) as well as the F_C3_ (*n* = 41) values by removing chemotypes with cannabinoid values of >90%/<10% and performed stepwise deletion of the data points with the largest standardized residuals (Figure 4B). For the di-/tri-cyclic cannabinoid fractions, three plants M01, M02, and M04 from the East Asian accession M contributed to reducing the explained variance between vegetative and maturation stages by 42.0% (Figures 4B,C), whereas for the C_3_-/C_5_-alkyl cannabinoid fractions, the removal of plants L13, L11 (L), and M05 (M) contributed negligibly to reducing the explained variance between vegetative and maturation stages (4.7%) (Figures 4B,C).

### Chemometric Categorization of Alkyl Cannabinoid Composition

**FIGURE 5:**
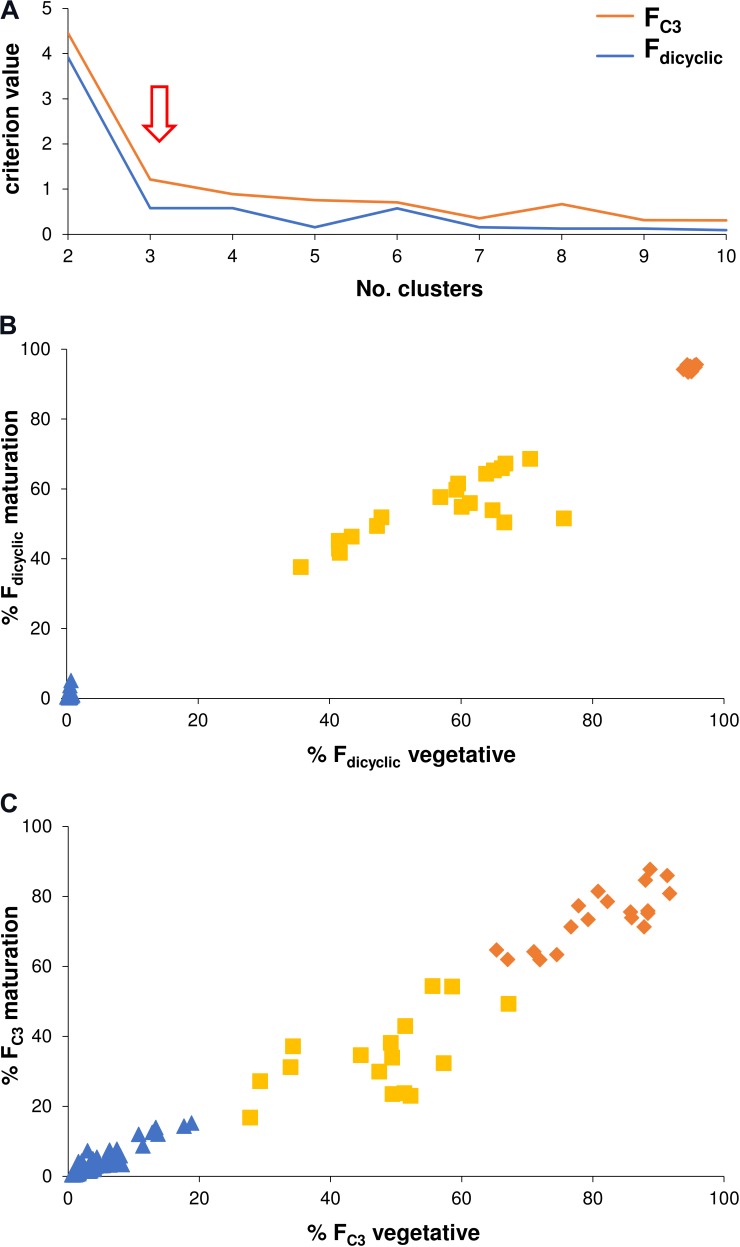
**(A)** Non-hierarchical *k*-means cluster analysis criterion values as a function of clusters. **(B)** Non-hierarchical *k*-means tripartite cluster analysis for the F_dicyclic_ chemotypic values across vegetative and maturation developmental stages. **(C)** Non-hierarchical *k*-means tripartite cluster analysis for the F_C3_ values across vegetative and maturation developmental stages. *Red arrow* indicates optimal number of clusters for the F_dicyclic_ as well as the F_C3_ chemotypic values; *Blue triangle* indicates low cannabinoid fraction cluster; *Yellow square* indicates intermediate cannabinoid fraction cluster; *Orange diamond* indicates high cannabinoid fraction cluster; C_3_-alkyl cannabinoid fractions (F_C3_); C_5_-alkyl cannabinoid fractions (F_C5_); and dicyclic cannabinoid fractions (F_dicyclic_).

Chemometric categorization of the di-/tri-cyclic as well as the C_3_-/C_5_-alkyl cannabinoid fractions was performed using non-hierarchical *k*-means cluster analysis which incorporated within-plant variation across vegetative, flowering and maturation developmental stages. This was based on the premise that the genotype does not vary over time, and that the continuity of the C_3_-/C_5_-alkyl cannabinoid fractions could be disentangled by removing non-genotypic contributions to chemotype. The optimal number of clusters based on criterion values as a function of clusters was the predicted three for the di-/tri-cyclic as well as three for the C_3_-/C_5_-alkyl cannabinoid fractions (Figure 5A). The categories of the F_dicyclic_ values formed from the cluster analysis were congruent with those determined from the F_dicyclic_: F_tricyclic_ frequency distributions (Figures 3B,C), with plants being categorized into low, intermediate and high F_dicyclic_ value classes (Figure 5B). For the F_C3_ values, plants were also categorized into low, intermediate and high classes (Figure 5C), with the F_C3_ clusters ranging between 0.43–22.81, 16.87–67.14, and 61.91–91.70%, respectively.

## Discussion

### Plasticity of Alkyl Cannabinoid Composition

The quantity and quality of secondary plant metabolites are often attributed to a combination of genetic and environmental (G x E) factors (Bustos-Segura et al., 2017), with chemotypic plasticity associated with changing expression patterns in response to biotic and abiotic cues (Wink, 2003). Under environmentally uniform conditions we found that the di-/tri-cyclic as well as the C_3_-/C_5_-alkyl cannabinoid fractions were relatively stable throughout development, which is consistent with previous reports of C_5_-alkyl cannabinoid composition from clonal (De Backer et al., 2012; Aizpurua-Olaizola et al., 2016) and seed propagated plants (Pacifico et al., 2008) grown in controlled environments. This suggests that the between-plant variation in cannabinoid quality observed within the diversity collection has a strong genetic influence independent of intragenerational environmental stimuli, and that the di-/tri-cyclic as well as the C_3_-/C_5_-alkyl cannabinoid chemotypes may have developed over longer periods via anthropogenic selective pressures and/or clinal adaptation. Indeed, intraspecific comparisons of *Artemisia californica* grown in a common environment together with precipitation manipulation treatments have shown limited plasticity in terpenoid quality, with compositional dissimilarity associated with source latitudinal distance (Pratt et al., 2014).

The between-plant alkyl cannabinoid chemotypic variation could have also been generated by the response of ecotypically distinct genotypes to a homogeneous environment. Understanding of how G × E interactions contribute to *in planta* cannabinoid quality is currently limited, and clonal analyses of ecotypes in response to temperature (Bazzaz et al., 1975), photoperiod (Valle et al., 1978) and other environmental cues are lacking. However, cannabinoid quality has been shown to be insensitive to environmental treatments such as ultraviolet (UV)-B radiation (Lydon et al., 1987). Quantitative polymerase chain reaction (qPCR) expression profiles of the genes *THCAS* (Sirikantaramas et al., 2004) and *CBDAS* (Taura et al., 2007) encoding the synthases responsible for stereospecific cyclisation of the major di-/tri-cyclic cannabinoids have also been poorly correlated to THCA (Cascini et al., 2013) and CBDA proportions (Onofri et al., 2015), while the presence or absence of functional *THCAS* and *CBDAS* genes has been found predictive of cannabinoid quality (Weiblen et al., 2015). Given that THCA:CBDA cannabinoid proportions typically follow Mendelian inheritance (De Meijer et al., 2003), and that crosses between high C_3_-alkyl cannabinoid inbreeds and a high C_5_-alkyl cannabinoid clone form F_1_ progenies with distinct C_3_-/C_5_-alkyl cannabinoid chemotypes intermediate to the parents (De Meijer and Hammond, 2016), a predominant genetic basis for cannabinoid quality is unambiguous.

Recent discoveries in the genomic organization of secondary plant metabolism genes and associated transcriptional regulatory mechanisms may provide explanation for the stability of the di-/tri-cyclic as well as the C_3_-/C_5_-alkyl cannabinoid fractions. The occurrence of non-homologous secondary metabolite gene clusters has been well documented in a number of diverse plant taxa (Boycheva et al., 2014). Chromatin immunoprecipitation analysis in *Arabidopsis thaliana* has shown that the histone variant H2A.Z facilitates localized nucleosome opening and expression of contiguous thalianol as well as marneral gene clusters, with independently formed clusters encoding product-specific oxidosqualene cyclases, cytochrome P450 enzymes and acyltransferases required for the synthesis of these triterpenoids (Nützmann and Osbourn, 2015). Despite limited characterization at all levels of gene cluster regulation, including analysis of promoter and *cis*-regulatory elements (Nützmann et al., 2016), evidence for the coordinated expressing of 43 secondary metabolic clusters has also been identified using the ATTED-II coexpression database (Aoki et al., 2016) in *A. thaliana, Sorghum bicolor, Oryza sativa*, and *Solanum lycopersicum* (Schläpfer et al., 2017).

It may be possible that the coordinated transcriptional regulation of non-homologous cannabinoid gene clusters limits expressional selectivity of cannabinoid pathway genes. This may result in increased stability of cannabinoid compositional homogeneity throughout development and limit variation in cannabinoid composition to heritable recombination events. While no direct observation of non-homologous gene clusters has yet been identified in *Cannabis*, evidence for tandem duplication of *THCAS* (McKernan et al., 2015) and potentially *CBDAS* (Onofri et al., 2015; Weiblen et al., 2015) as well as single gene transposition from long interspersed element-like (LINE-like) retrotransposons (Sakamoto et al., 2000) suggest that genomic reorganization mechanisms associated with metabolic gene cluster formation (Schläpfer et al., 2017) may have occurred. Completion of a fully annotated and chromosome-anchored genome assembly for *Cannabis* (Van Bakel et al., 2011; Vergara et al., 2016) may provide opportunities to elucidate the functional genomic architecture responsible for cannabinoid compositional stability. Functional characterization of alkyl-cannabinoid-determining loci may allow application of gene editing technologies, such as clustered regularly interspaced short palindromic repeats (CRISPR)/CRISPR-associated9 (Cas9) (Alagoz et al., 2016), for development of elite chemotypes capable of producing alkyl cannabinoids beyond that of C3 or C5 configurations (Vree et al., 1972; Smith, 1997). Genetic enhancement and precise metabolic engineering of the alkyl pharmacophoric element could not only lead to therapeutic cannabinoid portfolio expansion (De Meijer and Hammond, 2016), but may also facilitate quality improvement of plant-based cannabinoid production systems (Potter, 2014; Chandra et al., 2017).

### Chemotypic Heterozygosity

Heterozygosity at multiple chemotype-determining loci may account for a reduction of variance explained in the F_dicyclic_/F_tricyclic_ values between vegetative and maturation stages in a subset of East Asian individuals. Allelism tests on progenies segregating for THCA and CBDA support a co-dominant *B* locus model, whereby the alleles encoding THCA and CBDA synthase govern THCA:CBDA cannabinoid proportions (De Meijer et al., 2003). DNA marker analysis of *Cannabis* chemotypes has shown that F_dicyclic_/F_tricyclic_ values of ≥90% are associated with *THCAS* or *CBDAS* homozygosity, while intermediate chemotypes with F_dicyclic_/F_tricyclic_ values of <90% are associated with *THCAS* and *CBDAS* heterozygosity (Welling et al., 2016a). In the *THCAS*:*CBDAS* heterozygote state, functional synthases are believed to compete for the substrates CBGA and CBGVA (Shoyama et al., 1984). The catalytic efficiency of THCA and CBDA synthases are reported to be dependent on alkyl side chain length (Shoyama et al., 1984), which suggests that metabolic fluxes of CBGA or CBGVA substrate within a *THCAS*:*CBDAS* heterozygote individual could lead to transitional changes in the F_dicyclic_/F_tricyclic_ ratio.

To test whether the activity of THCA and CBDA synthase could be affected by CBGA or CBGVA substrates, we compared the F_dicyclic_/F_tricyclic_ values within the C_3_-/C_5_-alkyl cannabinoid fractions in mature *THCAS*:*CBDAS* heterozygote plants (*n* = 20). Despite a wide range of F_dicyclic_/F_tricyclic_ dissimilarity between the C_3_-/C_5_-alkyl cannabinoid fractions among genotypes, the F_C5_ F_dicyclic_/F_tricyclic_: F_C3_ F_dicyclic_/F_tricyclic_ ratio was 1.44 (±0.34%). Interestingly, the individuals M01, M02, and M04 which in the truncated chemotypic distribution contributed to developmental F_dicyclic_ variation, exhibited both the F_dicyclic_ and F_C3_ values close to parity at maturation, with M04 exhibiting F_dicyclic_ and F_C3_ values of 51.67% (±0.18%) and 54. 41% (±0.22%), respectively. Given that these individuals are likely *THCAS*: *CBDAS* heterozygotes which can produce both C_3_- and C_5_-alkyl cannabinoid precursors, substrate flux above either THCA or CBDA synthases’ *K_m_* could result in substrate competition that affects the steady state concentration and time-dependent behavior of cannabinoid end products (Schäuble et al., 2013), resulting in the non-conformity of the di-/tri-cyclic cannabinoid fractions observed between vegetative and maturative stages.

### Genetic Regulation of Alkyl Cannabinoid Composition

Despite the therapeutic importance of the cannabinoid alkyl side chain, the biosynthetic and genetic relationships responsible for alkyl homolog specificity remain poorly characterized in *Cannabis.* In the case of C_5_-alkyl cannabinoids, the prenylated resorcinyl core and alkyl side chain are formed from the fatty acid starter unit hexanoic acid. This undergoes cytosolic acyl-activation (Stout et al., 2012) as well as polyketide formation by a tetraketide synthase (TKS) and olivetolic acid cyclase (OAC) complex forming the alkylresorcinol olivetolic acid (Gagne et al., 2012), prior to aromatic prenylation by geranyl-pyrophosphate:olivetolate geranyltransferase (GOT) (Fellermeier and Zenk, 1998) forming CBGA.

A similar mechanism, involving butanoic acid as a starter unit and the alkylresorcinol divarinic acid, is predicted for the synthesis of CBGVA. This is based on the functional characterization of recombinant alkylresorcinol synthases in the Poaceae plant family, which utilize acyl-CoA variously to form alkylresorcinol side chain homologs (Cook et al., 2010), as well as TKS (Taura et al., 2009) and GOT (Page and Boubakir, 2011) accepting butanoyl-CoA and a variety of aromatic substrates, respectively. However, the origin and synthesis of hexanoic and butanoic acid are unknown (Marks et al., 2009; Stout et al., 2012), while understanding the contribution of intracellular compartmentation, including metabolon constructs, on the channeling, selection and utilization of cannabinoid precursors, is incomplete. Moreover, the enzymatic promiscuity or specificity of OAC (Gagne et al., 2012) and GOT (Page and Boubakir, 2011) has not been examined with the predicted C_3_-alkyl cannabinoid intermediates. Nonetheless, it appears plausible that changes in the alkyl side chain originate prior to and possibly at polyketide formation, implying that multiple loci contribute to C_3_-/C_5_-alkyl cannabinoid composition.

Allelism tests suggest that an oligogenic or polygenic multi-locus *A*^1^-*A*^2^-… *A*^n^ governs the C_3_-/C_5_-alkyl cannabinoid ratios in plants, although discontinuities in the C_3_-/C_5_-alkyl cannabinoid distributions of the available progeny were inadequate to form categorizations based on cannabinoid quality (De Meijer and Hammond, 2016). From the cluster analysis of within-plant variation, we identified three discrete F_C3_/F_C5_ categories (Figure 5C). As for the di-/tri-cyclic cannabinoid fractions (Figure 5B), the presence of three categories could indicate a monogenic model for C_3_-alkyl cannabinoid chemotypes, whereby allelic variation governing alkylresorcinol fatty acid starter unit availability or incorporation facilitates changes in the F_C3_/F_C5_ ratio. In a C_3_-/C_5_-alkyl cannabinoid monogenic model, small chemotypic differences between genotypes coupled with large individual variation within genotypic classes, could explain phenotypic continuity (Griffiths et al., 1999). However, the apparent absence of extreme individuals with F_C3_ values ≥90% within the sample population suggests the potential for additional categories, which would support an oligogenic or polygenic mechanism. In any case, the F_C3_/F_C5_ clusters identified are consistent with categorizations which can be expected within genetic resources of *Cannabis* and therefore offer utility in the selection and breeding of C_3_-alkyl cannabinoid genotypes.

As licit large-scale multi-billion dollar industries based on *Cannabis* emerge in the United States (Butsic et al., 2017), small incremental changes in the relative proportions of cannabinoids could have significant commercial and therapeutic implications for botanical drug development and manufacture (Potter, 2014; Chandra et al., 2017). Through selective inbreeding and hybrid clone selection, GW Pharmaceuticals, plc have reportedly achieved double- and triple-cross inbred plant lines with C_3_-alkyl cannabinoid proportions up to 96% (De Meijer and Hammond, 2016). In the current analysis we demonstrated a wide range of the C_3_-/C_5_-alkyl cannabinoid proportions within a relatively small subset of individuals from a single generation, which highlights the value of *Cannabis ex situ* conservation and characterization (Welling et al., 2016a). Comprehensive sampling of *Cannabis* genetic resources, both within and between accessions (Soler et al., 2017; Figure 3A), may make it possible to identify and select for pharmaceutically valuable chemotypes capable of reaching F_C3_ values ≥96%. However, it is uncertain whether the C_3_-alkyl cannabinoid fraction could match or exceed the C_5_-alkyl cannabinoid fraction in chemotypically extreme individuals. This may be affected by the lower molecular weight of C_3_-alkyl cannabinoid homologs which leads to a disproportionately reduced representation when comparing fractions/proportions derived from weight per weight concentrations.

## Conclusion

The major alkyl cannabinoids of *Cannabis* were characterized across three developmental stages within a chemotypic diversity panel. Under controlled conditions alkyl cannabinoid composition was found to be stable throughout development. This suggests a strong genotypic influence on alkyl cannabinoid compositional variation and the potential for genetic enhancement of the alkyl pharmacophoric element. Further chemical and genomic characterization of *Cannabis* genetic resources may provide greater insight into the genetic mechanisms responsible for alkyl cannabinoid composition and provide novel opportunities for the genetic metabolic engineering and pharmaceutical diversification of plant derived alkyl cannabinoids.

## Author Contributions

MW designed and performed the experiments and prepared the manuscript. LL provided contributions to conception and design of the research project, as well as development of analytical procedures, and provided detailed review and revision of the manuscript. CR performed statistical analyses and review and revision of the manuscript. OA provided background information and performed review and revision of the manuscript. GK provided substantial contributions to conception and design of the research project and performed detailed review and revision of the manuscript.

## Conflict of Interest Statement

Southern Cross University receives funding from the commercial entity Ecofibre Industries Operations Pty Ltd. Ananda Hemp Ltd are a subsidiary company of Ecofibre Industries Operations Pty Ltd. The authors declare that the research was conducted in the absence of any commercial or financial relationships that could be construed as a potential conflict of interest.

## Footnotes

1 www.gwpharm.com

2 anandahemp.com
