# Supplementary material for: Developmental Plasticity of the Major Alkyl Cannabinoid Chemotypes in a Diverse Cannabis Genetic Resource Collection
Source: Front Plant Sci. 2018 Oct 23;9:1510. doi: 10.3389/fpls.2018.01510 (PMC6206272; doi:10.3389/fpls.2018.01510)
Supplement: Supplementary file 1 [file Data_Sheet_1.docx]

***Supplementary Material***

**Developmental Plasticity of the Major Alkyl Cannabinoid Chemotypes in a Diverse Cannabis Genetic Resource Collection**

**Matthew T. Welling^1,2^ , Lei Liu^1^ , Carolyn A. Raymond^1^ , Omid Ansari^2,3^, Graham J. King^1,*^**

^1^Southern Cross Plant Science, Southern Cross University, Lismore, New South Wales 2480, Australia.

^2^Ecofibre Industries Operations Pty Ltd, Brisbane, Queensland 4014, Australia.

^3^Ananda Hemp Ltd, Cynthiana, Kentucky 41031, USA.

*** Correspondence:**Graham J. King
graham.king@scu.edu.au

**Supplementary Table S2.** Cannabinoid values for 99 *Cannabis* plants across three developmental stages

**Supplementary Figure S1.** LC-MS analysis of unknown compound **1**.

**Supplementary Figure S2.** LC-MS analysis of unknown compound **2**.

**Supplementary Figure S3.** ¹H NMR (800 MHz, DMSO-*d*_6_) spectrum of unknown compound **1**.^1^H NMR (DMSO-*d*_6_, 800 MHz) δ 13.6 (1H, bs, 1-OH), 13.6 (1H, bs, 2-COOH), 6.33 (1H, s, 10-H), 6.12 (1H, s, 4-H), 3.13 (1H, m, 6a-H), 2.70, 2.85 (2H, m, 1’-H), 2.10 (2H, m, 8-H), 1.87 (2H, m, 7-H), 1.62 (3H, bs, 11-CH_3_), 1.53 (1H, dt, *J* = 11.5, 2.0 Hz, 10a-H), 1.48 (2H, m, 2’-H), 1.37 (3H, s, 13-H), 1.02 (3H, s, 12-H), 0.87 (3H, t, *J* = 7.3 Hz, 3’-H)

**Supplementary Figure S4.** ¹H NMR (800 MHz, DMSO-*d*_6_) spectrum of unknown compound **2**. ^1^H NMR (DMSO-*d*_6_, 800 MHz) δ 9.81 (1H, bs, 1’-OH), 6.12 (1H, s,4’-H), 5.06 (1H, s, 2-H), 4.40, 4.45 (2H, m, 9-H), 3.87 (1H, m, 3-H), 3.02 (1H, m, 4-H), 2.73 (2H, bm, 1’’-H), 1.92, 2.10 (2H, m, 6-H), 1.61, 1.68 (2H, m, 5-H), 1.59 (3H, s, 7-H), 1.57 (3H, s, 10-H), 1.47 (2H, bm, 2’’-H), 0.86 (3H, t, *J* = 7.3 Hz, 3’’-H)

**Supplementary Figure S5.** ¹³C NMR (800 MHz, DMSO-*d*_6_) spectrum of unknown compound **1**.^13^C NMR (DMSO-*d*_6_, 800 MHz) δ 173.9 (COOH, 2-COOH), 164.1 (C, C-1), 158.0 (C, C-5), 133.4 (C, C-9), 133.2 (C, C-3), 124.2 (CH, C-10), 111.3 (CH, C-4), 109.7 (C, C-10b), 104.8 (C, C-2), 78.7 (C, C-6), 45.9 (CH, C-10a), 38.6 (CH_2_, C-1’), 33.6 (CH, C-6a), 31.3 (CH_2_, C-8), 27.3 (CH_3_, C-13), 25.0 (CH_2_, C-2’), 24.8 (CH_2_, C-7), 23.7 (CH_3_,C-11), 19.7 (CH_3_, C-12), 14.5 (CH_3_, C-3’)

**Supplementary Figure S6.** ¹³C NMR (800 MHz, DMSO-*d*_6_) spectrum of unknown compound **2**.^13^C NMR (DMSO-*d*_6_, 800 MHz) δ 174.2 (COOH, 2’-COOH), 160.2 (C,C-1’), 149.1 (C, C-8), 149.1 (C, C-5’), 144.6 (C, C-3’), 130.9 (C, C-1), 126.2 (CH, C-2), 114.8 (C, C-6’), 110.1 (CH_2_, C-9), 110.1 (CH, C-4’), 104.0 (C, C-2’), 43.6 (CH, C-4), 38.0 (CH_2_, C-1’’), 30.4 (CH_2_, C-6), 29.5 (CH_2_, C-5), 24.7 (CH_2_, 2’’), 23.6 (CH_3_,C-7), 19.2 (CH_3_, C-10), 14.4 (CH_3_, C-3’’)

**Supplementary Figure S7.** ^1^H-^1^H-COSY NMR (800 MHz, DMSO-*d*_6_) spectrum of unknown compound **1**.

**Supplementary Figure S8.** ^1^H-^1^H-COSY NMR (800 MHz, DMSO-*d*_6_) spectrum of unknown compound **2**.

**Supplementary Figure S9.** HSQC NMR (800 MHz, DMSO-*d*_6_) spectrum of unknown compound **1**.

**Supplementary Figure S10.** HSQC NMR (800 MHz, DMSO-*d*_6_) spectrum of unknown compound **2**.

**Supplementary Figure S11.** HMBC NMR (800 MHz, DMSO-*d*_6_) spectrum of unknown compound **1**.

**Supplementary Figure S12.** HMBC NMR (800 MHz, DMSO-*d*_6_) spectrum of unknown compound **2**.

**Supplementary Figure S13.** ROESY NMR (800 MHz, DMSO-*d*_6_) spectrum of unknown compound **1**.

**Supplementary Figure S14.** ROESY NMR (800 MHz, DMSO-*d*_6_) spectrum of unknown compound **2**.
