# Supplementary material for: Developmental Plasticity of the Major Alkyl Cannabinoid Chemotypes in a Diverse Cannabis Genetic Resource Collection
Source: Front Plant Sci. 2018 Oct 23;9:1510. doi: 10.3389/fpls.2018.01510 (PMC6206272; doi:10.3389/fpls.2018.01510)
Supplement: Supplementary file 2 [file Table_1.DOCX]

***Supplementary Material***

**Developmental Plasticity of the Major Alkyl Cannabinoid Chemotypes in a Diverse Cannabis Genetic Resource Collection**

**Matthew T. Welling^1,2^ , Lei Liu^1^ , Carolyn A. Raymond^1^ , Omid Ansari^2,3^, Graham J. King^1,*^**

^1^Southern Cross Plant Science, Southern Cross University, Lismore, New South Wales 2480, Australia.

^2^Ecofibre Industries Operations Pty Ltd, Brisbane, Queensland 4014, Australia.

^3^Ananda Hemp Ltd, Cynthiana, Kentucky 41031, USA.

*** Correspondence:**Graham J. King
graham.king@scu.edu.au

**Supplementary Table S1** LC-MS SIM mode setting for the detection of twelve cannabinoids.

| Signal | Time  (min) | Cannabinoid | SIM  ion | Fragmentor | Gain | Dwell  (ms) | % Rel  dwell |
| --- | --- | --- | --- | --- | --- | --- | --- |
| 1 | 1.8 | CBDV | 287 | 150 | 1.00 | 80 | 100.0 |
| 1 | 3.3 | CBD | 315 | 150 | 1.00 | 110 | 100.0 |
| 1 | 7.0 | THC | 315 | 150 | 1.00 | 110 | 100.0 |
| 1 | 9.4 | CBC | 315 | 150 | 1.00 | 110 | 100.0 |
| 2 | 2.6 | CBDA | 341 | 150 | 1.00 | 80 | 100.0 |
| 2 | 3.4 | THCV | 287 | 150 | 1.00 | 170 | 100.0 |
| 2 | 5.4 | CBN | 311 | 150 | 1.00 | 170 | 100.0 |
| 3 | 1.6 | CBDVA | 313 | 150 | 1.00 | 80 | 100.0 |
| 3 | 3.0 | CBGA | 343 | 150 | 1.00 | 80 | 100.0 |
| 4 | 3.2 | CBG | 317 | 150 | 1.00 | 80 | 100.0 |
| 4 | 4.6 | THCVA | 313 | 150 | 1.00 | 170 | 100.0 |
| 4 | 9.5 | THCA | 341 | 150 | 1.00 | 330 | 100.0 |

Allocation of cannabinoids over four selected-ion monitoring (SIM) signal channels for the quantification of cannabinoids. Signals 1-4 (% Cycle time, 25); Mode, SIM; Polarity, Positive. Cannabichromene (CBC); cannabidiol (CBD); cannabidiolic acid (CBDA); cannabidivarin (CBDV); cannabidivarinic acid (CBDVA); cannabigerol (CBG); cannabigerolic acid (CBGA); cannabinol (CBN); delta(9)-tetrahydrocannabinol (THC); delta(9)-tetrahydrocannabinolic acid (THCA); delta(9)-tetrahydrocannabivarin (THCV); delta(9)-tetrahydrocannabivarinic acid (THCVA)
